# Effects of Active Gamification on Sleep and Anxiety Reduction in Spanish Primary School Children

**DOI:** 10.3390/healthcare13060623

**Published:** 2025-03-13

**Authors:** María del Carmen Carcelén-Fraile, Alberto Ruiz-Ariza, Alba Rusillo-Magdaleno, Agustín Aibar-Almazán

**Affiliations:** 1 Department of Educational Sciences, Faculty of Social Sciences, University of Atlántico Medio, 35017 Las Palmas de Gran Canaria, Spain; carmen.carcelen@pdi.atlanticomedio.es; 2 Faculty of Educational Sciences, University of Jaén, 23071 Jaén, Spain; arusillo@ujaen.es; 3 Department of Health Sciences, Faculty of Health Sciences, University of Jaén, 23071 Jaén, Spain; aaibar@ujaen.es

**Keywords:** anxiety, sleep quality, gamification, exercise, primary education

## Abstract

**Background/Objectives**: Active gamification, which incorporates game elements with physical interaction, is presented as an innovative strategy to address anxiety problems and sleep quality in children. This study aimed to evaluate the impact of an active gamification program on the aforementioned variables in children in primary education. **Methods**: This study utilized a randomized, controlled trial with 120 children between 8 and 11 years of age, divided into an experimental group, which participated in a 12-week gamified program, and a control group, which continued with traditional physical education classes. **Results**: The main findings indicate that the intervention had a significant impact on reducing anxiety, with improvements in most of the subscales evaluated except for the obsessive-compulsive disorder subscale. Significant improvements were also found in sleep quality, with reductions in bedtime resistance, nighttime awakenings, parasomnias, and sleep-disordered breathing, although no significant changes were noted in sleep-onset delay, sleep duration, sleep anxiety, and daytime sleepiness. **Conclusions**: The gamification intervention in physical exercise showed positive effects in reducing anxiety and improving sleep quality in primary school children, highlighting its potential as an intervention strategy in primary education classrooms.

## 1. Introduction

The mental health of children in primary education has become a priority on the educational and social agenda [1]. Childhood, between the ages of 6 and 12, is a crucial stage for the comprehensive development of the individual, in which the foundations for mental health and well-being are established throughout life [2]. During these years, children experience significant changes at a cognitive, emotional, and social levels, and it is essential to provide them with an environment that favors their psychological and emotional development [3]. However, various factors can put children’s mental health at risk, such as family stress, learning difficulties, bullying, or exposure to violence [4].

According to data from the World Health Organization (WHO), it is estimated that between 10% and 20% of children and adolescents worldwide suffer from some mental disorder [5]. Specifically, in Spain, a significant prevalence of mental disorders has been observed in the child population. According to data from the Ministry of Health, 13.2% of children between 4 and 14 years of age are at risk of poor mental health, being more frequent in boys (15.6%) than in girls (10.5%). Furthermore, it has been reported that 20.8% of adolescents from 10 to 19 years of age suffer from some type of diagnosed mental health problem, which places Spain as the European country with the highest prevalence of mental disorders in young people [6]. These data underline the importance of addressing mental health from an early age to prevent future complications since inadequate management can negatively impact their academic, emotional, and social development [7].

Anxiety is one of the most common disorders in childhood and manifests itself through excessive worries and fears that can affect a child’s behavior, sleep, and eating [8]. In Spain, 10% of children in primary education suffer from anxiety, and this figure increases in children with learning difficulties or family problems [9]. At these ages, anxiety can manifest itself in various ways, such as difficulty concentrating, problems sleeping, irritability, headaches or stomach aches, and avoidance of social situations [10]. Therefore, it not only affects the child’s emotional well-being but can also have a negative impact on their academic performance and social relationships [11].

Sleep quality is a fundamental factor that influences children’s mental health and general well-being [12]. During sleep, the brain not only processes and consolidates learned information but also regulates essential functions for emotional and cognitive development [13]. Neuronal activity during nighttime rest favors the maturation of brain circuits that are key in emotional regulation, memory, and decision making [14]. In particular, slow-wave sleep (NREM) plays a fundamental role in synaptic plasticity and consolidation of learning, while the REM phase has been linked to stress regulation and emotional modulation [15]. Poor sleep quality can have negative consequences for children’s health. At the cognitive level, it has been documented that the lack of adequate rest can affect concentration, reduce the ability to solve problems, impair working memory, and decrease academic performance [16]. In the emotional and behavioral realm, children who experience sleep disturbances have higher levels of irritability, impulsivity, and difficulties in emotional regulation, which can increase the likelihood of developing behavioral problems [17]. In addition, various studies have shown that the lack of sleep is associated with an increased risk of psychiatric disorders throughout childhood and adolescence, including anxiety, depression, and attention deficit hyperactivity disorder (ADHD) [18,19]. Sleep disorders in childhood can manifest in various ways, each with specific implications for children’s emotional and behavioral well-being. Among the most common is childhood insomnia, characterized by difficulties in falling or staying asleep, which may be associated with separation anxiety, night terrors, or inadequate sleep hygiene habits [20]. Frequent nighttime awakenings also affect sleep continuity, which can lead to daytime sleepiness and chronic fatigue [21]. Sleep disturbances can affect brain circuits related to emotional control and stress regulation, increasing vulnerability to psychological problems and affecting the quality of life of children and their families [22]. Anxiety and sleep disorders have been shown to have a bidirectional relationship, whereby elevated levels of anxiety can lead to sleep disturbances, while sleep deprivation or poor-quality sleep can increase vulnerability to anxiety and other emotional problems [23]. Specifically in childhood, anxiety is strongly associated with problems falling asleep and frequent night awakenings [24]. Similarly, children with anxiety present longer sleep latency and lower sleep efficiency [25]. Regarding sleep quality, poor sleep quality in childhood has been shown to directly affect emotional well-being and stress regulation, which can lead to an increase in anxious symptoms [26]. On the other hand, the literature has pointed out that sleep deprivation can be a significant risk factor for the onset and maintenance of anxiety in children and adolescents [27]. Sleep fragmentation and lack of adequate rest can amplify negative emotional responses and reduce coping capacity in stressful situations [28]. It has been established that school-aged children should sleep between 9 and 12 h a day to ensure optimal development. However, currently, a significant percentage of the child population does not reach this recommended rest time [29]. In Spain, approximately 30% of children do not sleep the appropriate number of hours for their age, which increases the prevalence of sleep problems such as insomnia, night terrors, and frequent awakenings, problems that not only affect their immediate well-being but can also have a long-term impact on their mental health, contributing to the development of anxiety symptoms and difficulties in emotional self-regulation [30]. This evidence supports the hypothesis that interventions aimed at improving sleep quality can have positive effects on reducing anxiety, which reinforces the relevance of the present study.

Physical exercise plays a fundamental role in improving sleep quality and reducing anxiety in children [31]. Physical activity releases endorphins, neurotransmitters that have a positive effect on mood and help reduce stress and anxiety levels, promoting better mental health in children [32] and more restful and deeper sleep [33]. The WHO recommends that children aged 5 to 17 years perform at least 60 min of moderate or vigorous physical activity daily [34]. However, various studies have indicated that the rate of childhood sedentary lifestyle has increased in recent years, which may be negatively influencing sleep quality and the appearance of anxiety symptoms [35,36]. Therefore, schools should implement strategies that encourage physical exercise both inside and outside the classroom, promoting healthy lifestyle habits from an early age [37]. The integration of physical activity in the educational context not only contributes to the physical health of children but also has a positive impact on their emotional well-being and the prevention of mental disorders [38].

In this context, gamification in physical education classes is presented as an effective strategy to encourage children’s adherence and motivation towards physical activity [39]. This consists of the integration of game elements, such as rewards, challenges, level progression, and competitive or cooperative dynamics, in order to make exercise more attractive and stimulating [40]. This approach transforms physical activity into a playful experience, encouraging the active participation of students and promoting more meaningful learning [41]. From a psychological perspective, gamification enhances intrinsic motivation, which favors the continuity of sports practice and reduces social anxiety in the classroom [42]. Furthermore, the combination of exercise and game elements stimulates the release of endorphins and dopamine, neurotransmitters that contribute to the regulation of mood and a reduction in stress levels [32]. In physiological terms, it has been shown that children aged 8 to 11 years who participate in gamified activities have a better quality of sleep since physical activity facilitates the regulation of the circadian rhythm and reduces the time needed to fall asleep [41]. This study reinforces the importance of gamification as an educational tool. Furthermore, previous research in Spain has used similar methodologies in the field of physical education and child health, obtaining results aligned with those of this study [32,35]. In short, gamification not only improves the experience of physical exercise but also optimizes its benefits on mental health and child well-being. Its implementation in the school environment can represent a key tool for promoting healthy lifestyle habits, reducing anxiety symptoms, and improving the quality of students’ sleep, thus promoting their comprehensive development [42].

For all these reasons, the present study aimed to evaluate the effectiveness of an active gamification program, as implemented in the physical education classroom, on anxiety and sleep quality in primary education students. In accordance with the objective of this study, it was hypothesized that the implementation of an active gamification program in physical education classes would be effective in reducing anxiety and improving sleep quality in primary education students.

## 2. Materials and Methods

### 2.1. Study Design

A randomized, controlled clinical trial was conducted to evaluate the effects of a gamification program on anxiety and sleep quality in primary school children. This study was approved by the Ethics Committee of the University of Atlántico Medio (CEI01-014), and its design complies with Spanish legislation regarding clinical research with human beings (Law 3/5 December 2018, on Biomedical Research), the regulations on the protection of personal data (Organic Law 15/1999), and the principles established in the 2013 version of the Declaration of Helsinki, held in Brazil.

### 2.2. Participants

**Figure 1:**
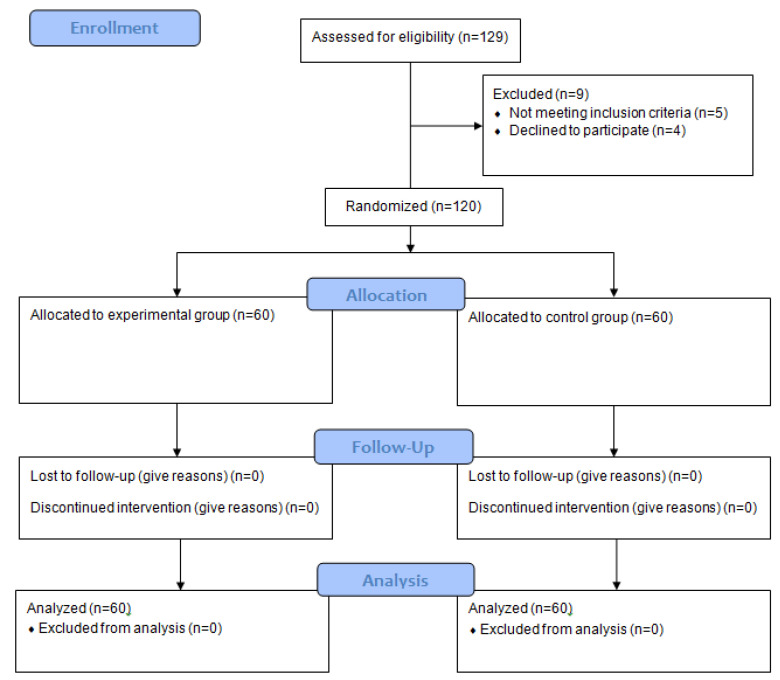
Flowchart of participants in the study.

Of the 129 students initially recruited, 120 were included in the study because they met the following inclusion criteria: (i) age between 8 and 11 years; (ii) no medical contraindication for physical activity; (iii) no intellectual disability that could interfere with understanding or compliance with the study procedures; and (iv) informed consent from parents or legal guardians (Figure 1). Those students who did not meet the inclusion criteria were able to participate in the intervention with their peers but were not considered in the analysis. The participants selected for the study had a mean age of 6.67 ± 1.02 years, with 58.50% boys and 41.50% girls.

### 2.3. Measures

#### 2.3.1. Anxiety

Anxiety was assessed using the brief version of the Spence Children’s Anxiety Scale for Spanish children (SCAS-C-8) [43] in its version validated in Spanish [44], which consists of 18 items grouped into 6 subscales: (i) separation anxiety disorder (SAD): 5, 8, and 12; (ii) social phobia (SoP): 2, 6, and 10; (iii) obsessive-compulsive disorder (OCD): 9, 13, and 14; (iv) physical injury fears (PIF): 3, 7, and 11; (v) generalized anxiety disorder (GAD): 1, 4, and 15; (vi) panic disorder (PD): 16, 17, and 18; and (vii) total score, which is obtained by adding the scores of the 18 items. Each item is rated on a Likert-type scale from 0 to 3: 0 = never, 1 = sometimes, 2 = often, and 3 = always. The total score ranges from 0 to 54 points, with higher scores indicating a greater presence of anxiety symptoms. The reliability for this sample had a Cronbach’s α value of 0.755.

#### 2.3.2. Sleep Quality

The Children’s Sleep Habits Questionnaire (CSHQ) is a widely used questionnaire to assess the perception of sleep quality in children [45]. For this study, the validation for Spanish children was used [46]. It consists of 33 items, each with a Likert response scale from 1 to 3: 1 = never (0–1 time per week), 2 = sometimes (2–4 times per week), and 3 = frequently (5–7 times per week), with a total score ranging from 33 to 99 points. Higher scores indicate greater sleep problems. This scale is grouped into eight subscales, each assessing a different aspect of sleep: (i) bedtime resistance: 1, 3, 4, 5, 6, and 8; (ii) sleep-onset delay: 2; (iii) sleep duration: 9 and 10; (iv) sleep anxiety: 5, 7, 11, and 27; (v) night wakings: 23 and 24; (vi) parasomnias: 12, 13, 14, 15, 16, 17, 18, and 19; (vii) sleep-disordered breathing: 25, 26, and 28; (viii) daytime sleepiness: 20, 21, 22, 29, 30, 31, 32, and 33; and (ix) CSHQ global score, which is the sum of the 33 items. The reliability for this sample was a Cronbach’s α value of 0.772.

### 2.4. Intervention

The experimental group (EG) participated in a gamification intervention in physical exercise aimed at primary school children, with 45 min sessions three times a week. Each session was organized into 10 min of warm-up (including activation activities such as reaction games or mini-challenges to prepare children physically and mentally), 30 min dedicated to the main phase of the activity, and 5 min for awards and updating group rankings. The lesson design in this study was based on the principles of instructional design, ensuring an organized and effective intervention. According to theoretical models such as Gagné’s Instructional Design [47] and Keller’s ARCS Model [48], effective teaching should include strategies that promote attention, motivation, and knowledge retention. In this line, the gamification-based program was conceived with the purpose of stimulating students’ active participation and enriching their learning experience by integrating game mechanics into physical activity. Previous research has shown that the application of gamification in educational environments can increase student motivation and engagement [49,50]. Considering these principles, the program structured the lessons incorporating essential elements such as well-defined learning objectives, immediate feedback, and progressive challenges tailored to the individual abilities of the students. The program consisted of three thematic phases with increasing intensity and complexity:

Phase 1: Explorers in Action (weeks 1–4). (A) Week 1 was the introduction to exploration, including teamwork and space exploration activities, such as the game “Conquering Territories”, where children had to complete physical tasks to earn points. Coordination and planning were also worked on through “Bridges and Rivers”, and agility and endurance with “Explorer’s Footprints”. (B) Week 2 consisted of coordination challenges with games such as “Building Towers” and “Treasure Cave” that worked on coordination, agility, and collaboration. Speed and endurance were reinforced through “Explorer’s Race”. (C) Week 3 involved overcoming explorer challenges utilizing the games “Explorer’s Maze” and “Hunting for Lost Objects”, which focused on agility, endurance, observation, and teamwork. (D) Week 4 involved consolidating the explorer, in which children completed speed, agility, and teamwork challenges, such as “Treasure Hunt” and “Explorer’s Challenge”, which integrated all the skills developed so far.

Phase 2: Movement Heroes (Weeks 5–8). (A) Week 5 was hero training, in which children developed stamina and coordination with games like “Hero’s Journey” and “Defending the Fortress”. (B) Week 6 was kingdom rescue; Games like “Quest for the Gems” and “Road to the Kingdom” promoted collaboration and strategy skills. (C) Week 7 involved advanced collaboration challenges, with activities like “Resource Quest” and “Protecting the Fortress” that challenged children in areas like planning and teamwork. (D) Week 8 was the heroes’ final challenge, and this final week included combined stamina and collaboration challenges like “The Final Battle” and “Dragon Race”.

Phase 3: Health Guardians (weeks 9–12). (A) Week 9 involved guardian challenges, where children worked on strength, coordination, and teamwork through games such as “Knocking Down Enemy Towers” and “Obstacle Relay”. (B) Week 10 comprised guardian missions, with challenges such as “Searching for Lost Resources” and “Strategic Transfer” fostering problem solving and team collaboration. (C) Week 11 involved preparing for the final battle; endurance and planning sessions such as “Team Endurance Race” and “Final Battle Simulation” consolidated the acquired skills. (D) Week 12 was the final battle, and the final session, “The Grand Final Battle”, integrated all the skills the students worked on in a circuit of stations, where teams competed in various physical tests.

In this way, the program was designed to ensure constant progress in physical skills and teamwork, gradually increasing the difficulty and complexity of the challenges.

### 2.5. Procedure

For the selection of participants, contact was established with a school located in the province of Jaén (Spain). A meeting was scheduled with the parents or legal guardians of the students in order to provide information about the study, giving them an informational document and the corresponding informed consent. Only students whose parents or guardians gave their authorization participated in the study. The children were randomly assigned to one of two study groups: an EG, which participated in a program based on the gamification of physical exercise, and a control group (CG), which continued with their traditional physical education classes. The assignment was carried out using a procedure of sealed, sequentially numbered envelopes that were opened by a person external to the study, guaranteeing the masking of the process. It was carried out in a 1:1 ratio, using a computer-generated random number table. Neither the participants nor the researchers nor the teachers knew the group assignment. Questionnaires were administered to the students before and after the intervention to evaluate the study variables. The teachers in charge of implementing both the gamification program and the traditional physical education classes received detailed instructions before the start of the intervention. In the case of the experimental program, the teachers participated in a training on the gamification methodology for two weeks prior to the start of the sessions. This training was key to ensuring that the activities were developed in accordance with the established design principles. During the intervention, the researchers carried out continuous monitoring and supervision of the classes. In addition, observations were carried out to verify compliance with the activities and the stipulated times as well as the active participation of the students, who were required to attend all the sessions of the program. Weekly checklists were used to monitor compliance with the guidelines, and an assessment of adherence was carried out through direct observation of the intervention sessions. Since factors such as physical activity performed by students outside of school hours could influence the study variables, additional monitoring was implemented through periodic telephone calls to the parents of participants in both groups. These calls were intended to verify that students did not perform extracurricular physical activity. The activities were carried out during school hours, from 12:00 to 12:45.

### 2.6. Data Analysis

Means and standard deviations were calculated for all variables of interest. Differences between groups were assessed using the Student’s *t*-test for independent samples. Before performing the analyses, the normality of distributions and homogeneity of variances were checked using the Kolmogorov–Smirnov and Levene tests, respectively. To analyze the effect of the intervention, a repeated-measures analysis of variance (ANOVA) with a mixed design was used. The between-subjects factor was the intervention group (EG vs. CG), while the within-subjects factor corresponded to the time of assessment (pre- and post-intervention assessments). Dependent variables included sleep quality and anxiety, and specific tests were performed for each. The possible interaction between group assignment and time of assessment was also examined. Effect sizes were calculated using Cohen’s *d*, considering thresholds of ≤0.2 for small effects, 0.5 for moderate effects, and 0.8 for large effects. Significance was set at *p* < 0.05. To analyze the relationships between variables after the intervention, Pearson correlation coefficients were calculated between the post-intervention measures, differentiating between the experimental group and the control group. Correlations with a *p*-value < 0.05 were considered statistically significant. All analyses were performed using SPSS software version 17.0 (SPSS, Inc., Chicago, IL, USA).

## 3. Results

No significant differences were observed between the CG and the EG in relation to anxiety and sleep quality at pre-intervention measurements (all *p* > 0.05) (Table 1). Students attended a minimum of 98.3% of the scheduled intervention sessions, and no incidents related to injuries or adverse reactions were recorded throughout the intervention period.

### 3.1. Anxiety

**Figure 2:**
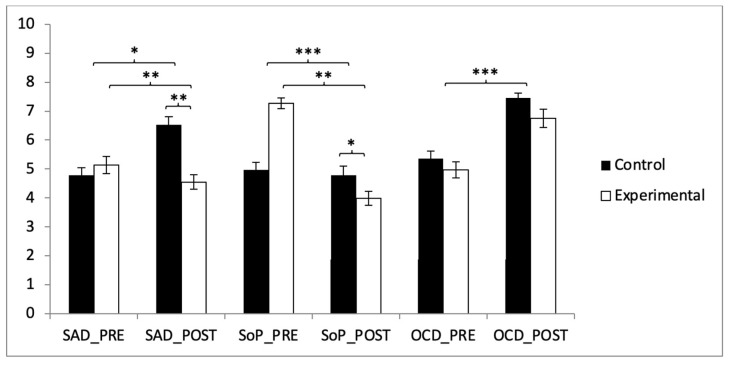
Inter- and intragroup comparisons regarding anxiety: * *p* < 0.05; ** *p* < 0.01; *** *p* < 0.001.

Our analysis revealed that participants in the experimental group exhibited improvements in their scores for SAD, SoP, PIF, GAD, PD, and SCAS-C-8 after the intervention (Table 2). Specifically, for the SAD subscale, significant differences were observed between the two groups in the post-intervention assessment (t(118) = 5.387, *p* < 0.001, with a Cohen’s *d* of 0.98). Additionally, statistically significant differences were found between pre- and post-intervention scores within the experimental group (t(59) = −6.649, *p* < 0.001, and Cohen’s *d* of 0.27) (Figure 2).

In the SoP subscale, a subsequent analysis of the interaction revealed significant differences between the two groups in the post-intervention measurement (t(118) = 2.024, *p* = 0.045, with a Cohen’s *d* of 0.37). Statistically significant differences were also observed between pre- and post-intervention results in the experimental group (t(59) = −6.649, *p* < 0.001, with a Cohen’s *d* of 1.96). However, no significant improvements were found in the OCD subscale (Table 2 and Figure 2).

**Figure 3:**
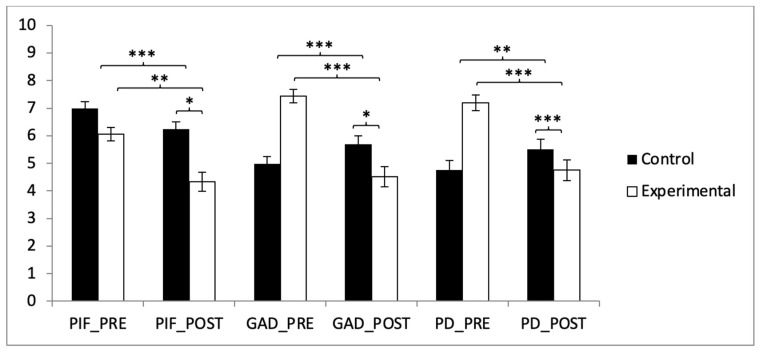
Inter- and intragroup comparisons regarding anxiety: * *p* < 0.05; ** *p* < 0.01; *** *p* < 0.001.

In the PIF subscale, the analysis showed significant differences between the two groups in the post-intervention measurement (t(118) = 4.166, *p* < 0.001, with a Cohen’s *d* of 0.76). Statistically significant differences were also observed between pre- and post-intervention results in the experimental group (t(59) = 4.651, *p* < 0.001, with a Cohen’s *d* of 0.76). In the GAD subscale, a subsequent analysis of the interaction revealed significant differences between the two groups in the post-intervention measurement (t(118) = 2.359, *p* = 0.020, with a Cohen’s *d* of 0.06). Statistically significant differences were also observed between pre- and post-intervention results in the experimental group (t(59) = 6.912, *p* < 0.001, with a Cohen’s *d* of 1.25). In the PD subscale, the results showed significant differences between the two groups in the post-intervention measurement (t(118) = 1.366, *p* < 0.001, with a Cohen’s *d* of 0.06). Statistically significant differences were also observed between pre- and post-intervention results in the experimental group (t(59) = 6.912, *p* < 0.001, with a Cohen’s *d* of 1.25) (Figure 3).

**Figure 4:**
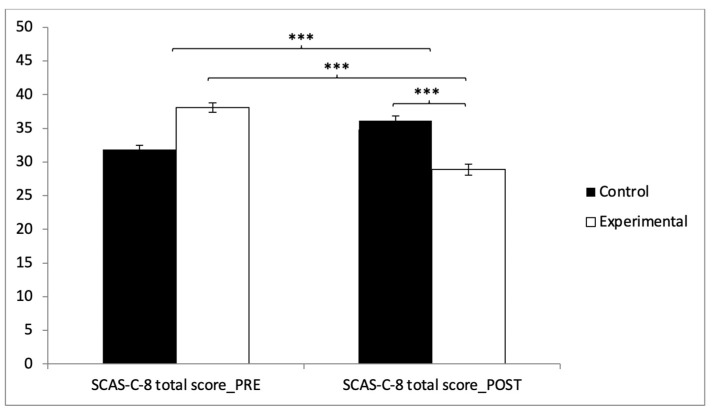
Inter- and intragroup comparisons regarding total score of anxiety: *** *p* < 0.001.

Finally, for the SCAS-C-8 total score, the results showed significant differences between the two groups in the post-intervention measurement (t(118) = 6.817, *p* < 0.001, with a Cohen’s *d* of 1.25). Statistically significant differences were also observed between pre- and post-intervention results in the experimental group (t(59) = 9.212, *p* < 0.001, with a Cohen’s *d* of 1.55) (Figure 4).

### 3.2. Sleep Quality

**Figure 5:**
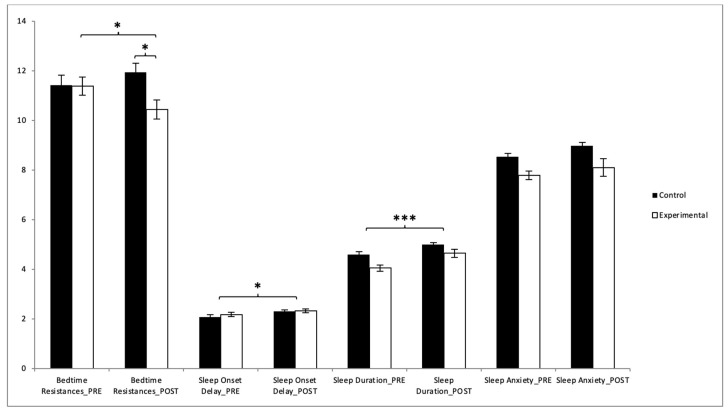
Inter- and intragroup comparisons regarding total score of sleep quality: * *p* < 0.05; *** *p* < 0.001.

Our analysis showed that participants in the EG saw improvements in their scores for bedtime resistance, night wakings, parasomnias, sleep-disordered breathing, and the CSHQ global score after the intervention period (Table 2). Regarding the bedtime resistances, a subsequent analysis of the interaction revealed that there were significant differences between the two groups in the post-intervention measurement (t(118) = 2.808, *p* = 0.006, with a Cohen’s *d* of 0.51). Statistically significant differences were also observed between pre- and post-intervention results in the experimental group (t(59) = 2.141, *p* = 0.0036, with a Cohen’s *d* of 0.33) (Figure 5). However, no significant improvements were found in the sleep-onset delay, sleep duration, and sleep anxiety.

**Figure 6:**
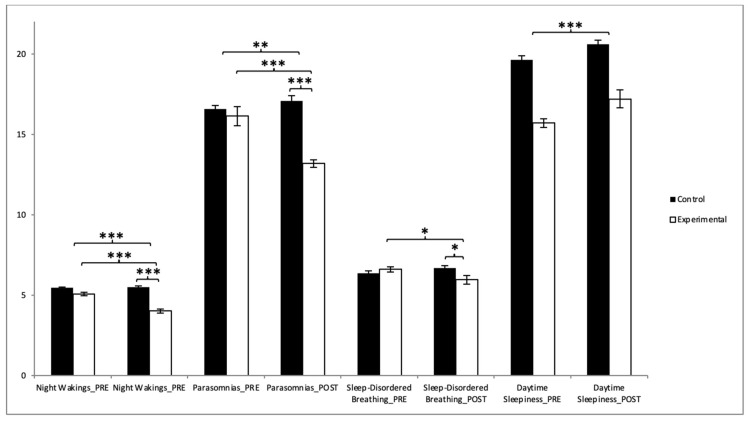
Inter- and intragroup comparisons regarding total score of sleep quality: * *p* < 0.05; ** *p* < 0.01; *** *p* < 0.001.

In the case of night wakings, the results showed significant differences between the two groups in the post-intervention measurement (t(118) = 9.740, *p* = 0.000, with a Cohen’s *d* of 0.78). Statistically significant differences were also observed between pre- and post-intervention results in the experimental group (t(59) = 5.705, *p* = 0.000, with a Cohen’s *d* of 1.13). In the parasomnias, the results showed significant differences between the two groups in the post-intervention measurement (t(118) = 9.827, *p* = 0.000, with a Cohen’s *d* of 0.79). Statistically significant differences were also observed between pre- and post-intervention results in the experimental group (t(59) = 5.705, *p* = 0.000, with a Cohen’s *d* of 0.83). In the sleep-disordered breathing, the results showed significant differences between the two groups in the post-intervention measurement (t(118) = 2.309, *p* = 0.023, with a Cohen’s *d* of 0.42). Statistically significant differences were also observed between pre- and post-intervention results in the experimental group, (t(59) = 2.458, *p* = 0.017, with a Cohen’s *d* of 0.37) (Figure 6). However, no significant improvements were found in daytime sleepiness (Table 2).

**Figure 7:**
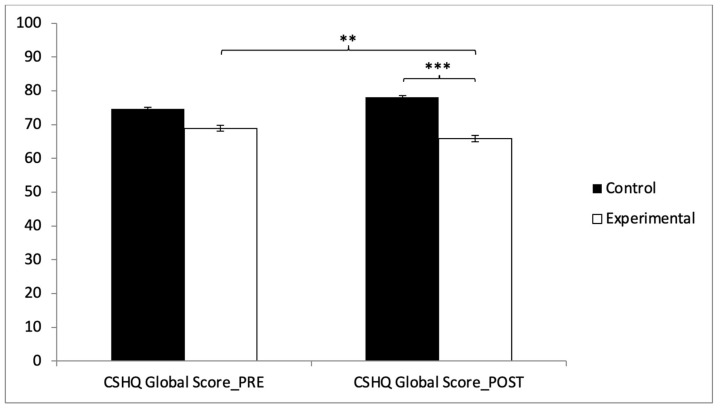
Inter- and intragroup comparisons regarding total score of sleep quality: ** *p* < 0.01; *** *p* < 0.001.

In the CSHQ global score, the results showed significant differences between the two groups in the post-intervention measurement (t(118) = 11.464, *p* = 0.000, with a Cohen’s *d* of 1.09). Statistically significant differences were also observed between pre- and post-intervention results in the experimental group (t(59) = 3.197, *p* = 0.002, with a Cohen’s *d* of 0.47) (Figure 7).

## 4. Discussion

The aim of the present study was to evaluate the effects of a gamification intervention in physical exercise on anxiety and sleep quality in primary school children. The main findings indicate that the intervention had a significant impact on reducing anxiety, with improvements in most of the subscales evaluated except for the obsessive-compulsive disorder subscale. Significant improvements were also found in sleep quality, with reductions in bedtime resistance, nighttime awakenings, parasomnias, and sleep-disordered breathing, although no significant changes were noted in sleep-onset delay, sleep duration, sleep anxiety, and daytime sleepiness.

Anxiety in childhood is a determining factor in socio-emotional development, and its impact on long-term mental health has been widely documented [51]. In our research, we observed significant reductions in several anxiety subscales after the intervention. In the SAD, our findings showed a significant decrease in the experimental group because physical exercise has been associated with a reduction in separation anxiety due to its impact on cortisol regulation and stress response [52]. These results coincide with previous studies that, although they carried out interventions different from our study, showed improvements in this type of anxiety, such as the study by Comer et al. [53], in which they carried out a psychoeducational intervention in very young children.

In the SoP subscale, our results also reflect a significant decrease. Physical exercise, especially in group settings, has been shown to be effective in improving social confidence and reducing social anxiety [54]. In our study, this physical exercise is carried out through gamification, which not only promotes physical activity in a playful and motivating way but also favors social interaction and engagement with the intervention. Previous research has found a reduction in social anxiety in children, such as the study by Scaini et al. [55], a comprehensive meta-analysis of cognitive–behavioral interventions (CBT) whose results supported the efficacy of CBT in reducing the severity of social anxiety symptoms in both clinical and school settings and supported the claim that incorporating social skills training into programs is beneficial.

Regarding the PIF subscale, our results show significant improvements, which coincides with previous studies, such as that by Morgado et al. [56], which highlighted the effectiveness of psychoeducation in emotional regulation in reducing intolerance to uncertainty but, unlike our study, in adolescents aged 10 to 19 years. Additionally, it has been shown that physical activity can improve tolerance to uncertainty, largely by decreasing the physiological hyperactivation associated with stress and anxiety [57]. These findings underscore the importance of interventions that combine emotional work with physical strategies, providing a comprehensive approach to managing uncertainty.

In the GAD subscale, our intervention through gamification of physical exercise showed a significant reduction in symptoms. This result is consistent with previous studies indicating that the combination of cognitive restructuring techniques and progressive muscle relaxation improves generalized anxiety, especially in children [58]. In our case, gamification offered a playful way to integrate physical exercise, which favored engagement and adherence, contributing to better emotional regulation. Furthermore, physical exercise, through the release of neurotransmitters such as serotonin and GABA, has been shown to reduce generalized anxiety [59], and gamification amplifies these effects by creating an interactive environment that promotes motivation and reduces the perception of effort.

This finding is consistent with previous research that has shown that interventions combining physical exercises, especially when gamified, have a positive impact on reducing generalized anxiety in children. Recent studies have shown that gamification of physical exercise improves not only motivation and adherence to programs but also emotional regulation, which contributes significantly to the reduction in anxiety symptoms in children [60,61]. These innovative approaches, by integrating physical activity with playful elements, facilitate coping with anxiety and improve emotional well-being in the child population.

Sleep quality is a crucial factor in child development, influencing key areas such as emotional regulation, academic performance, and overall well-being [62]. In this study, the results indicate significant improvements in several dimensions of sleep following the gamification-based exercise intervention, especially in bedtime endurance, nighttime awakenings, and parasomnias. These results are consistent with the literature suggesting that regular physical activity can improve sleep quality and that incorporating playful and motivational elements, such as those used in gamification, can increase adherence to exercise programs, which positively impacts sleep patterns [63,64]. In terms of bedtime resilience, children who participated in the intervention showed significant improvements, suggesting that physical exercise, especially when presented in a playful and engaging way through gamification, may help regulate circadian rhythms and facilitate a smoother transition to bedtime. This improvement in sleep resilience is consistent with previous studies indicating that physical activity supports the regulation of the sleep–wake cycle and may help reduce anxiety related to bedtime [65]. Gamification, by incorporating extrinsic motivation and immediate rewards, may facilitated continue participation in physical activity, contributing to these benefits.

Regarding nighttime awakenings and parasomnias, a significant improvement was also observed in the experimental group. Gamification in physical exercise, by encouraging regular participation and enjoyment of the activity, seems to have helped reduce nighttime awakenings, probably due to its impact on emotional regulation and reducing physiological arousal before sleep. These findings are consistent with previous research suggesting that moderate physical exercise can reduce the frequency of nighttime awakenings and improve sleep efficiency [27,66]. Regarding sleep-disordered breathing, the results show an improvement, suggesting that the benefits of physical activity could also extend to improving nighttime breathing. While sleep-disordered breathing has a complex etiology, some studies have indicated that exercise can positively influence breathing and reduce sleep apnea episodes, which could be explained by improved emotional regulation and relaxation [67].

Despite improvements observed in several sleep dimensions, no significant improvements were found in sleep latency, sleep duration, sleep-related anxiety, and daytime sleepiness. These results suggest that although gamification in physical exercise has a positive impact on several aspects of sleep, some factors, such as sleep-related anxiety and disturbances in more complex sleep patterns, may be more resistant to this type of intervention. Previous studies have pointed out that sleep-related anxiety as well as chronic sleep disorders may require more specific interventions, such as cognitive–behavioral therapy, to achieve significant improvements [68]. Furthermore, factors such as biological predisposition, family dynamics, and preexisting sleep routines may influence the persistence of certain sleep problems regardless of interventions.

Limitations of our study include the lack of long-term follow-up to assess the persistence of intervention effects. Furthermore, reliance on self-reports may introduce biases in the perception of changes. Future research should consider the use of objective sleep measures such as actigraphy and the inclusion of longitudinal follow-ups to assess the durability of observed effects.

## 5. Conclusions

The results of this study suggest that a gamified physical exercise intervention may be an effective strategy to reduce anxiety and improve sleep quality in primary school children. The improvements observed in general anxiety and in several aspects of sleep, such as bedtime resistance and frequency of night awakenings, indicate that this approach may be beneficial in educational and family settings. These implications are particularly valuable since anxiety and sleep disorders are common problems in childhood that can affect children’s emotional and physical well-being. The implementation of gamified physical exercise programs could be an accessible and attractive way to improve mental health and sleep quality in this population. However, the results also indicate that significant changes were not evident in certain specific domains, suggesting the need to continue exploring the mechanisms underlying these effects. Furthermore, it is important to note that individual factors may influence the response to the program, highlighting the need to personalize the intervention according to the characteristics of the participants to maximize its effectiveness.

## Figures and Tables

**Table 1:** Preintervention sociodemographic characteristics of the participants.

		Total (*n* = 120)	Experimental (*n* = 60)	Control (*n* = 60)	*p*-Value
Age		9.40 ± 0.98	9.25 ± 0.93	9.55 ± 1.02	0.183
Sex	Boy	53 (44.20)	26 (49.10)	27 (50.90)	0.719
	Girl	67 (55.80)	34 (50.70)	33 (49.30)	
Weight (kg)		30.08 ± 2.82	29.65 ± 2.73	30.51 ± 2.87	0.262
Height (m)		1.32 ± 0.04	1.32 ± 0.04	1.33 ± 0.03	0.523
BMI (kg/m^2^)		17.12 ± 0.78	17.01 ± 0.70	17.22 ± 0.84	0.165
Mother’s studies (%)	No studies	6 (5.00)	4 (66.70)	2 (33.30)	0.258
	Primary studies	17 (14.20)	7 (41.20)	10 (58.80)	
	Secondary studies	46 (38.30)	20 (43.50)	26 (56.50)	
	University studies	51 (42.50)	29 (56.90)	22 (43.10)	
SAD		4.96 ± 2.17	5.13 ± 2.33	4.78 ± 1.99	0.189
SoP		6.12 ± 2.09	7.27 ± 1.47	4.97 ± 1.99	0.061
OCD		5.17 ± 2.08	4.97 ± 2.20	5.37 ± 1.95	0.318
PIF		6.52 ± 2.04	6.07 ± 1.91	6.97 ± 2.08	0.159
GAD		6.20 ± 2.38	7.43 ± 1.87	4.97 ± 2.19	0.070
PD		5.97 ± 2.87	7.20 ± 2.21	4.73 ± 2.95	0.175
SCAS-C-8 total score		34.92 ± 6.17	38.07 ± 5.32	31.78 ± 5.33	0.793
Bedtime resistances		11.39 ± 3.04	11.38 ± 2.81	11.40 ± 3.27	0.086
Sleep-onset delay		2.12 ± 0.75	2.18 ± 0.73	2.07 ± 0.78	0.768
Sleep duration		4.32 ± 1.00	4.05 ± 0.98	4.58 ± 0.96	0.811
Sleep anxiety		8.15 ± 1.28	7.78 ± 1.32	8.52 ± 1.13	0.087
Night qakings		5.27 ± 0.70	5.08 ± 0.77	5.45 ± 0.57	0.210
Parasomnias		16.35 ± 3.50	16.13 ± 4.65	16.57 ± 1.72	0.500
Sleep-disordered breathing		6.50 ± 1.19	6.62 ± 1.30	6.38 ± 1.06	0.088
Daytime sleepiness		17.66 ± 2.81	15.70 ± 2.03	19.62 ± 2.01	0.916
CSHQ global score		71.76 ± 6.20	68.93 ± 6.29	74.58 ± 4.68	0.058

Quantitative variables are presented as mean and standard deviation. Qualitative variables are presented as frequency and percentage. SAD: separation anxiety disorder; SoP: social phobia; OCD: obsessive-compulsive disorder; PIF: physical injury fears; GAD: generalized anxiety disorder; PD: panic disorder; SCAS-C-8: The Spence Children’s Anxiety Scale for Spanish; CSHQ: Children’s Sleep Habits Questionnaire.

**Table 2:** Effects of gamification on anxiety and sleep quality.

	EG (*n* = 60)		CG (*n* = 60)		Group			Time			Group × Time		
	Pre	Post	Pre	Post	F(90)	*p*-Value	η^2^	F(90)	*p*-Value	η^2^	F(90)	*p*-Value	η^2^
SAD	5.13 ± 2.33	4.55 ± 1.98	4.78 ± 1.99	6.53 ± 2.05	7.857	0.006	0.062	5.552	0.020	0.045	22.209	0.000	0.158
SoP	7.27 ± 1.47	3.98 ± 1.86	4.97 ± 1.99	4.78 ± 2.43	7.842	0.016	0.062	52.318	0.000	0.307	41.836	0.000	0.262
OCD	4.97 ± 2.20	6.75 ± 2.44	5.37 ± 1.95	7.45 ± 1.32	4.743	0.031	0.039	51.648	0.000	0.304	0.311	0.578	0.003
PIF	6.07 ± 1.91	4.33 ± 2.63	6.97 ± 2.08	6.22 ± 2.31	17.587	0.000	0.130	23.398	0.000	0.183	4.139	0.044	0.034
GAD	7.43 ± 1.87	4.68 ± 2.50	4.97 ± 2.19	4.52 ± 2.90	3.414	0.067	0.028	17.833	0.000	0.131	48.640	0.000	0.292
PD	7.20 ± 2.21	4.52 ± 2.90	4.97 ± 2.19	4.68 ± 2.50	4.229	0.042	0.035	9.101	0.003	0.072	32.248	0.000	0.215
SCAS-C-8 total score	38.07 ± 5.32	28.88 ± 6.47	31.78 ± 5.33	36.15 ± 5.13	0.363	0.548	0.003	15.511	0.000	0.116	122.750	0.000	0.510
Bedtime resistances	11.38 ± 2.81	10.43 ± 2.98	11.40 ± 3.27	11.93 ± 2.88	2.613	0.190	0.022	0.558	0.457	0.005	7.069	0.009	0.057
Sleep-onset delay	2.18 ± 0.73	2.33 ± 0.60	2.07 ± 0.78	2.28 ± 0.64	0.777	0.380	0.007	4.865	0.029	0.040	0.161	0.689	0.001
Sleep duration	4.05 ± 0.98	4.65 ± 1.26	4.58 ± 0.96	4.98 ± 0.75	9.587	0.000	0.973	17.807	0.000	0.131	0.712	0.400	0.006
Sleep anxiety	7.78 ± 1.32	8.10 ± 2.74	8.52 ± 1.13	8.95 ± 1.23	12.105	0.001	0.093	2.921	0.090	0.024	0.071	0.791	0.001
Night wakings	5.08 ± 0.77	4.02 ± 1.08	5.45 ± 0.57	5.52 ± 0.50	114.922	0.000	0.493	21.139	0.000	0.152	27.151	0.000	0.187
Parasomnias	16.13 ± 4.65	13.18 ± 1.92	16.57 ± 1.72	17.08 ± 2.40	36.917	0.000	0.238	9.468	0.003	0.074	19.217	0.000	0.140
Sleep-disordered breathing	6.62 ± 1.30	5.97 ± 2.08	6.38 ± 1.06	6.68 ± 1.20	1.288	0.259	0.011	1.165	0.283	0.010	8.583	0.004	0.068
Daytime sleepiness	15.70 ± 2.03	17.20 ± 4.31	19.62 ± 2.01	20.60 ± 2.01	81.770	0.000	0.409	16.589	0.000	0.123	0.718	0.398	0.006
CSHQ global score	68.93 ± 6.29	65.88 ± 6.80	74.58 ± 4.68	78.03 ± 4.60	102.870	0.000	0.466	0.132	0.717	0.001	34.834	0.000	0.228

Quantitative variables are presented as mean and standard deviation. EG: experimental group; CG: control group; SAD: separation anxiety disorder; SoP: social phobia; OCD: obsessive-compulsive disorder; PIF: physical injury fears; GAD: generalized anxiety disorder; PD: panic disorder; SCAS-C-8: The Spence Children’s Anxiety Scale for Spanish; CSHQ: Children’s Sleep Habits Questionnaire.

## Data Availability

The data presented in this study are available on request from the corresponding author. The data are not publicly available because, due to the sensitive nature of the questions asked in this study, participants were assured raw data would remain confidential and would not be shared.

## Abbreviations

The following abbreviations are used in this manuscript:

WHO	World Health Organization
SCAS-C-8	The Spence Children’s Anxiety Scale for Spanish children
SAD	Separation anxiety disorder
SoP	Social phobia
OCD	Obsessive-compulsive disorder
PIF	Physical injury fears
GAD	Generalized anxiety disorder
PD	Panic disorder
CSHQ	The Children’s Sleep Habits Questionnaire
EG	Experimental group
CG	Control group
CBT	Cognitive–behavioral interventions
